# Crystal structure and Hirshfeld surface analysis of tris­(2,2′-bi­pyridine)­nickel(II) bis­(1,1,3,3-tetra­cyano-2-eth­oxy­propenide) dihydrate

**DOI:** 10.1107/S2056989019006959

**Published:** 2019-05-24

**Authors:** Ignacio Chi-Duran, Zouaoui Setifi, Fatima Setifi, Christian Jelsch, Bernd Morgenstern, Andres Vega, Felipe Herrera, Dinesh Pratap Singh, Kaspar Hegetschweiler, Rabab Boyaala

**Affiliations:** a Department of Physics, Universidad of Santiago Chile, Av. Ecuador 3493, Estaciín Central, Santiago 9170124, Chile; b Millennium Institute for Research in Optics, MIRO, Chile; c Département de Technologie, Faculté de Technologie, Université 20 Août 1955-Skikda, BP 26, Route d’El-Hadaiek, Skikda 21000, Algeria; d Laboratoire de Chimie, Ingénierie Moléculaire et Nanostructures (LCIMN), Université Ferhat Abbas Sétif 1, Sétif 19000, Algeria; e Cristallographie, Résonance Magnétique et Modélisations (CRM2), UMR CNRS 7036, Institut Jean Barriol, Université de Lorraine, BP 70239, Boulevard des Aiguillettes, 54506 Vandoeuvre-les-Nancy, France; f Fachrichtung Chemie, Universität des Saarlandes, Postfach 151150, D-66041 Saarbrücken, Germany; g Departamento de Ciencias Quimicas, Universidad Nacional Andres Bello, Av Republica 275 3er Piso, Santiago, Region Metropolitana, Chile; h Laboratoire de Chimie Appliquée et Environnement (LCAE), Faculté des Sciences, Université Mohamed Premier, BP 524, 60000, Oujda, Morocco

**Keywords:** crystal structure, polynitrile ligand, Hirshfeld surface analysis, hydrogen bonding

## Abstract

The title compound, [Ni(C_10_H_8_N_2_)_3_](C_9_H_5_N_4_O)_2_·2H_2_O, crystallizes as a racemic mixture in the monoclinic space group *C*2/*c*. In the crystal, the 1,1,3,3-tetracyano-2-ethoxypropenide anions and the water molecules are linked by O—H⋯N hydrogen bonds, forming chains running along the [010] direction. The bpy ligands of the cation are linked to the chain *via* C—H⋯π(cation) inter­actions involving the CH_3_ group. The inter­molecular inter­actions were investigated by Hirshfeld surface analysis and two-dimensional fingerprint plots.

## Abstract 2

The title compound crystallizes as a racemic mixture in the monoclinic space group *C*2/*c*. In the crystal, the 1,1,3,3-tetracyano-2-ethoxypropenide anions and the water molecules are linked by O—H⋯N hydrogen bonds, forming chains running along the [010] direction. The bpy ligands of the cation are linked to the chain *via* C—H⋯π(cation) inter­actions involving the CH_3_ group.

## Chemical context   

The use of polynitrile anions as ligands, either alone or in combination with neutral co-ligands, is a very promising and appealing strategy to obtain mol­ecular architectures with different topologies and dimensionalities owing to their ability to coordinate and bridge metal ions in many different ways (Miyazaki *et al.*, 2003 ▸; Atmani *et al.*, 2008 ▸; Benmansour *et al.*, 2007 ▸, 2008 ▸; Yuste *et al.*, 2009 ▸; Gaamoune *et al.*, 2010 ▸; Addala *et al.*, 2015 ▸; Setifi *et al.*, 2010 ▸, 2013*a* ▸,*b* ▸, 2014*a* ▸,*b* ▸, 2015 ▸, 2016 ▸, 2017 ▸). The presence of several potentially coordinating nitrile groups, their rigidity and their electronic delocalization, allows the synthesis of original magnetic high-dimensional coordination polymers with transition-metal ions (Benmansour *et al.*, 2010 ▸).
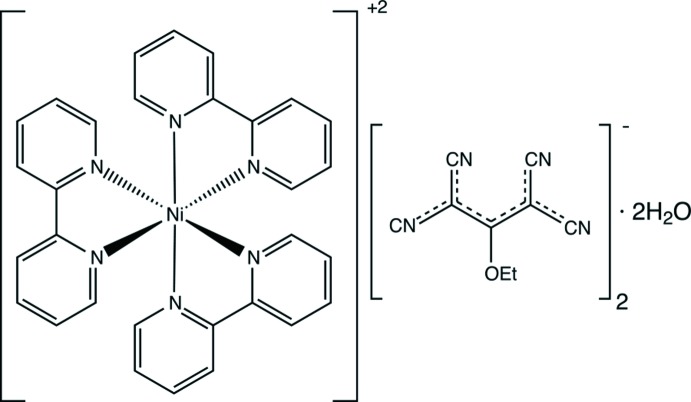


In view of the possible roles of these versatile polynitrile ligands, we have been inter­ested in using them in combination with other chelating or bridging neutral co-ligands to explore their structural and electronic characteristics in the field of mol­ecular materials exhibiting inter­esting magnetic exchange coupling. During the course of attempts to prepare such complexes with 2,2-dipyridyl, we isolated the title compound, whose structure is described herein along with the Hirshfeld surface analysis.

## Structural commentary   

**Figure 1:**
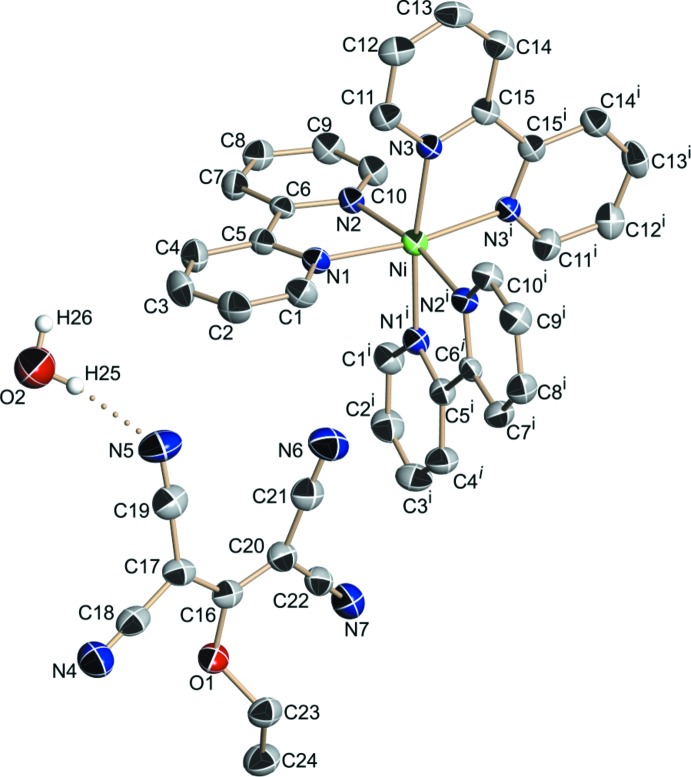
Mol­ecular structure of the title compound, showing the atom labeling and displacement ellipsoids drawn at the 50% probability level. Hydrogen atoms on the [Ni(bpy)_3_]^2+^ cation and (tcnoet)^−^ were omitted for clarity. Symmetry code: (i) 1 − *x*, *y*, 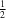 − *z*.

**Figure 2:**
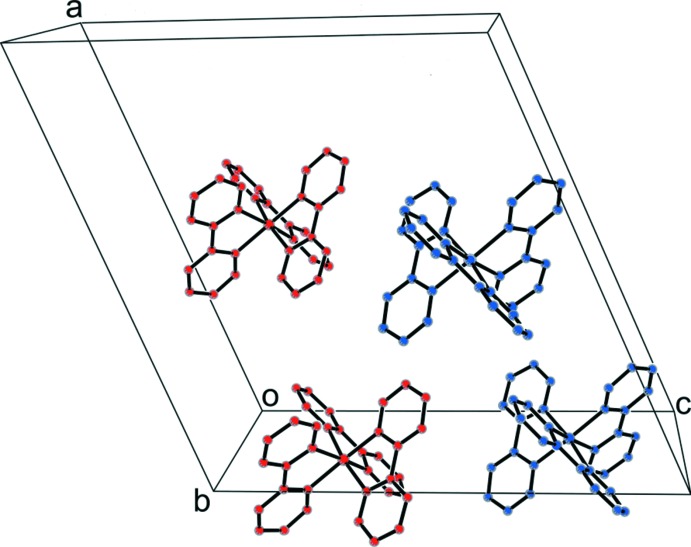
Disposition of Δ (red) and Λ (blue) stereoisomers in the unit cell.

The asymmetric unit of the title compound comprises a half of [Ni(bpy)_3_]^2+^ cation, one (tcnoet)^−^ anion and a solvent water mol­ecule within the monoclinic *C*2/*c*-centred cell (Fig. 1 ▸). In addition, this compound crystallizes presenting Δ and Λ chiral configurations and related to each other by inversion, forming a racemic mixture as illustrated in Fig. 2 ▸; this compound is isostructural to Fe(bpy)_3_(tcnoet)_2_(2H_2_O) (Setifi *et al.*, 2014*c* ▸). The Ni atom is located on the Wyckoff position 4*e* on the twofold axis. The [Ni(bpy)_3_]^2+^ complex presents a slightly distorted octa­hedral geometry of *C*_2_ point-group symmetry (Table 1 ▸). The Ni—N bond lengths are very similar to each other, being in the range 2.077 (3)–2.090 (3) Å, which is in agreement with the Ni—N distances for other [Ni(bpy)_3_]^2+^ complexes reported in the literature (Freire *et al.*, 2000 ▸; Su *et al.*, 2007 ▸; Yang *et al.*, 1998 ▸). In addition, the Ni—N distances are slightly longer than the Fe—N bonds [Fe(bpy)_3_]^2+^ [1.971 (2)–1.972 (2) Å] because of the larger Ni^2+^ radius compared to Fe^2+^ in a low-spin configuration (Shannon & Prewitt, 1969 ▸). The distorted N—Ni—N angles of the chelating bi­pyridine ligands [78.26 (16)–78.64 (12)°] are significantly less than 90°, as is usually found for [Ni(bpy)_3_]^2+^ complexes (Freire *et al.*, 2000 ▸; Yang *et al.*, 1998 ▸).

## Supra­molecular features   

**Figure 3:**
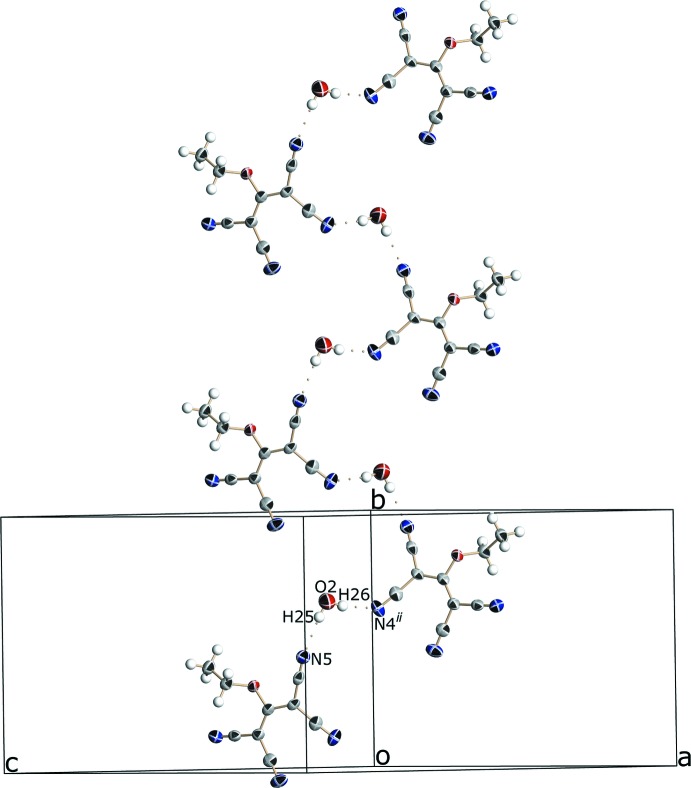
Partial crystal packing diagram showing the alternating zigzag (tcnoet)^−^–water chains defined by O—H⋯N hydrogen bonds running along the [010] direction. Symmetry code: (ii) −*x* + 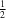, *y* − 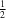, −*z* + 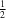.

As shown in Fig. 2 ▸, there are four [Ni(bpy)_3_]^2+^ cationic units within the unit cell of the compound, charge-balancing the1,1,3,3-tetra­cyano-2-eth­oxy­propenide anions. These, together with the hydration water, define planar and zigzag hydrogen-bonded chains, in which anions and water mol­ecules alternate, running along the [010] direction, as shown in Fig. 3 ▸. The O(water)—H⋯N(cyano) hydrogen-bonding inter­actions (Table 2 ▸) define the chain, with H⋯N distances of 2.11 and 2.10 Å. Finally, a C—H⋯π inter­action between the CH_3_ group of the (tcnoet)^−^ anion and the bpy ligand is observed, with a H⋯centroid distance of 3.01 Å (Table 2 ▸).

## Hirshfeld surface analysis   

**Figure 4:**
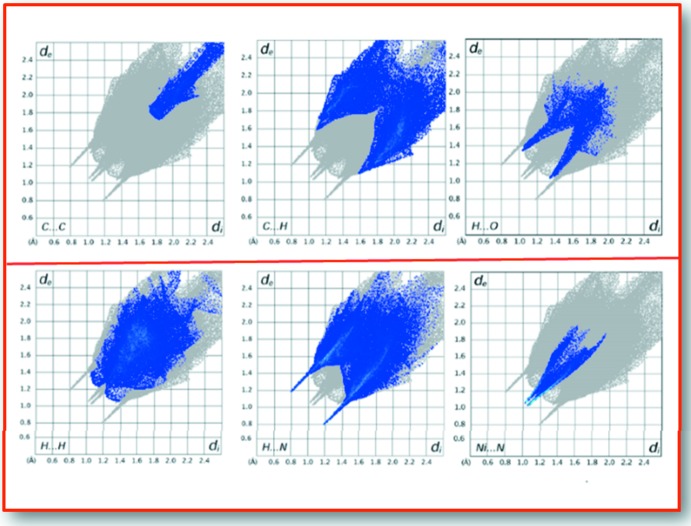
Hirshfeld surface fingerprint plot for the title compound showing the C⋯C, C⋯H, H⋯O, H⋯H, H⋯N and Ni⋯N contacts in detail.

The fingerprint plots (Fig. 4 ▸) of the inter­molecular contacts were computed using program *CrystalExplorer* (McKinnon *et al.*, 2007 ▸; Wolff *et al.*, 2012 ▸). The short contacts spikes are due to the N⋯H hydrogen bonds (outer spikes) and to the Ni⋯N coordination bonds (inner spikes).

The proportions of the different contacts and their enrichment (Jelsch *et al.*, 2014 ▸; Table 3 ▸) were computed with program *MoProViewer* (Guillot *et al.*, 2014 ▸). The enrichment ratios *E_x_*_y_ are obtained from the actual contacts between the different chemical species (*x*, *y*) and equi-probable proportions computed from the surface chemical content (Jelsch *et al.*, 2014 ▸). They allow contacts that are favored (over-represented) and which are likely to be the crystal driving force to be highlighted.

The Hirshfeld surface was computed for all the entities present in the crystal – the (tcnoet)^−^ anion, the [Ni(bpy)_3_]^2+^ complex and the water mol­ecule – in order to analyze the crystal contacts. Moieties not in contact with each other were selected in the crystal packing in order to obtain integral surfaces.

The nickel cation does not contribute to the mol­ecular surface, as it is coordinated by six nitro­gen atoms within the [Ni(bpy)_3_]^2+^ complex. Nearly three quarters of the Hirshfeld surface is of hydro­phobic in nature, constituted by atoms C and Hc. The most abundant contact is of the C⋯Hc type as a result of the extensive C—H⋯π inter­actions involving the aromatic rings. The second major contact is N⋯Hc, which is due to the abundance of the N and Hc chemical types and to the significant enrichment of this favorable weak hydrogen bond. The third major contact is of the C⋯C type and is due to stacking between the [Ni(bpy)_3_]^2+^ aromatic rings and the C(C(C≡N)_2_)_2_ group of the (tcnoet)^−^ anion.

The other significantly over-represented contacts are the strong hydrogen bonds N⋯H—O (*E* = 2.5) between the water mol­ecule and two nitrile groups. These are the hydrogen bonds with shortest distance *d*(N5⋯H25) = 2.11 Å and *d*(N4⋯H26) = 2.10 Å (Table 1 ▸). There is a deficit of strong hydrogen-bond donors compared to acceptors in this crystal structure. As a result, weak hydrogen bonds to H—C groups are formed. N⋯H—C weak hydrogen bonds occur and are slightly enriched. The oxygen atoms form only weak O⋯H—C hydrogen bonds, which are quite favored at *E* = 1.8. Globally there are two O—H⋯N strong hydrogen bonds, six C—H⋯N and two C—H⋯O weak hydrogen bonds (Table 2 ▸). The two major hydro­phobic contacts, C⋯H*c* and C⋯C, are both slightly enriched. If all hydro­phobic contacts (within C and H*c* atoms) are considered together, they are globally slightly under-represented with an enrichment ratio *E* = 0.92 because of the avoidance of the less favorable H*c*⋯H*c* contacts. All contacts between charged atoms (O, H*o*, N) are absent except for the attractive N⋯H*o* hydrogen bond. The cross hydro­philic/hydro­phobic contacts are slightly over-represented at *E* = 1.16 because of the occurrence of many weak O⋯H*c* and N⋯H*c* hydrogen bonds, which result from an unbalanced number of strong hydrogen-bond acceptors *versus* donors.

## Database survey   

The Cambridge Structural Database (CSD, Version 5.39, update August 2018, Groom *et al.*, 2016 ▸) includes a few structures involving polycyano­propide counter-ions, of which only 16 entries are hexa­cyano derivatives and four have (tcnoet)^−^ anions. There are no significant differences in C—N and C—C bond lengths between the hexa­cyano derivatives and (tcnoet)^−^ anions. However, the C21—C20—C16—C17 torsion angles in (tcnoet)^−^ anion (15.78°) are slightly smaller than the analogous torsion angle in other anions (16.32–21.68°). This difference can be explained by this compound and its isostructural structure featuring two hydrogen bonds, O2—H25⋯N5 and O2—H25⋯N4^ii^. These inter­actions orient the cyano groups toward to coplanarity with respect to other (tcnoet)^−^ mol­ecules that exhibit fewer hydrogen bonds. Finally, this compound has been used for the synthesis of low-dimensional metal–organic frameworks employing Mn^II^, Cu^II^, Co^II^ and Fe^II^ ions because the half cyano groups inter­act by hydrogen bonding with the metal aqua complexes, avoiding the formation of high-dimensional frameworks (Thétiot *et al.*, 2003 ▸).

## Synthesis and crystallization   

The title compound was synthesized solvothermally under autogenous pressure from a mixture of Ni(NO_3_)_2_·6H_2_O (29 mg, 0.1 mmol), 2,2-dipyridyl (16 mg, 0.1 mmol) and K(tcnoet) (45 mg, 0.2 mmol) in water–ethanol (4:1 *v*/*v*, 20 cm^−3^). This mixture was sealed in a Teflon-lined autoclave and held at 423 K for three days, and then cooled to ambient temperature at a rate of 10 K h^−1^ (yield: 54%). Light-green blocks of the title compound suitable for single-crystal X-ray diffraction were selected directly from the synthesized product.

## Refinement   

Crystal data, data collection and structure refinement details are summarized in Table 4 ▸. All H atoms were located in difference-Fourier maps. C-bound H atoms were then treated as riding atoms: C—H = 0.95 Å (aromatic), 0.98 Å (CH_3_) or 0.99 Å (CH_2_), and with *U*_iso_(H) = *kU*_eq_(C), where *k *= 1.5 for the methyl groups, which were permitted to rotate but not to tilt, and 1.2 for all others. H atoms bonded to the water O atom were permitted to ride at the positions located in the difference map, with *U*_iso_(H) = 1.5*U*_eq_(O).

## Supplementary Material

Crystal structure: contains datablock(s) I. DOI: 10.1107/S2056989019006959/mw2141sup1.cif

Structure factors: contains datablock(s) I. DOI: 10.1107/S2056989019006959/mw2141Isup2.hkl

CCDC reference: 1915961

Additional supporting information:  crystallographic information; 3D view; checkCIF report

## Figures and Tables

**Table 1:** Selected geometric parameters (Å, °)

Ni—N2	2.077 (3)	Ni—N3	2.090 (3)
Ni—N1	2.088 (3)		
			
N1—Ni—N2	78.64 (12)	N2—Ni—N3	92.87 (11)
N1^i^—Ni—N2	93.73 (11)	N1—Ni—N3	95.55 (11)
N1—Ni—N1^i^	91.20 (16)	N2—Ni—N3^i^	95.52 (11)
N1—Ni—N3^i^	171.36 (11)	N3—Ni—N3^i^	78.26 (16)
N2—Ni—N2^i^	169.18 (16)		

Symmetry code: (i) 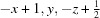.

**Table 2:** Hydrogen-bond geometry (Å, °) *Cg*1 is the centroid of the N2/C6–C10 ring.

*D*—H⋯*A*	*D*—H	H⋯*A*	*D*⋯*A*	*D*—H⋯*A*
O2—H25⋯N5	0.86	2.11	2.945 (5)	164
O2—H26⋯N4^ii^	0.86	2.10	2.955 (5)	175
C24—H24*A*⋯*Cg*1^iii^	0.98	3.01	3.921	156

Symmetry codes: (ii) 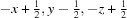; (iii) 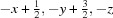.

**Table 3:** Nature of inter­molecular contacts on the Hirshfeld surface by chemical type The top part of the table gives the contribution *S*_*x*_ of each chemical type *X* to the Hirshfeld surface. The chemical types are grouped as hydro­phobic (C, H*c*) and charged (N, H*o*, O) atoms. The next part shows the percentage contributions *C*_*xy*_ of the actual contact types to the surface. The lower part of the table shows the *E*_*xy*_ contact enrichment ratios. The major *C*_*xy*_ contact types and the *E*_*xy*_ ratios much larger than unity (enriched contacts) are highlighted in bold. The hydro­phobic H*c* atoms bound to carbon are distinguished from the more polar H*o* water hydrogen atoms.

Atom type	H*o*	O	N	H*c*	C
Surface (%)	5.3	4.5	16.5	38.0	35.7
Ho	0.0				
O	0.0	0.0			
					
Contacts (%)					
N	**5.0**	0.0	0.0		
Hc	4.7	6.1	**20.9**	7.5	
C	1.9	3.0	8.5	**27.8**	**14.7**
Ho	0.0				
O	0.0	0.0			
					
Enrichment					
N	**2.5**	0.0	0.0		
Hc	1.1	**1.8**	**1.6**	0.54	
C	0.47	0.92	0.7	1.06	1.2

**Table 4:** Experimental details

Crystal data	
Chemical formula	[Ni(C_10_H_8_N_2_)_3_](C_9_H_5_N_4_O)_2_·2H_2_O
*M*_r_	933.63
Crystal system, space group	Monoclinic, *C*2/*c*
Temperature (K)	162
*a*, *b*, *c* (Å)	20.345 (3), 12.439 (3), 19.575 (4)
β (°)	112.800 (9)
*V* (Å^3^)	4566.8 (17)
*Z*	4
Radiation type	Mo *K*α
μ (mm^−1^)	0.49
Crystal size (mm)	0.17 × 0.14 × 0.07

Data collection	
Diffractometer	Bruker APEXII CCD
Absorption correction	Multi-scan (*SADABS*; Bruker, 2012 ▸)
*T*_min_, *T*_max_	0.683, 0.745
No. of measured, independent and observed [*I* > 2σ(*I*)] reflections	22477, 4670, 2483
*R*_int_	0.108
(sin θ/λ)_max_ (Å^−1^)	0.627

Refinement	
*R*[*F* ^2^ > 2σ(*F* ^2^)], *wR*(*F* ^2^), *S*	0.061, 0.125, 1.00
No. of reflections	4670
No. of parameters	310
No. of restraints	2
H-atom treatment	H atoms treated by a mixture of independent and constrained refinement
Δρ_max_, Δρ_min_ (e Å^−3^)	0.36, −0.40

Computer programs: *APEX2* and *SAINT* (Bruker, 2012 ▸), *SHELXS97* and *SHELXTL* (Sheldrick, 2008 ▸), *SHELXL2014* (Sheldrick, 2015 ▸) and *publCIF* (Westrip, 2010 ▸).

## supplementary crystallographic information

### Crystal data

**Table:**

[Ni(C_10_H_8_N_2_)_3_](C_9_H_5_N_4_O)_2_·2H_2_O	*F*(000) = 1936
*M**_r_* = 933.63	*D*_x_ = 1.358 Mg m^−^^3^
Monoclinic, *C*2/*c*	Mo *K*α radiation, λ = 0.71073 Å
*a* = 20.345 (3) Å	Cell parameters from 1400 reflections
*b* = 12.439 (3) Å	θ = 2.5–19.1°
*c* = 19.575 (4) Å	µ = 0.49 mm^−^^1^
β = 112.800 (9)°	*T* = 162 K
*V* = 4566.8 (17) Å^3^	Block, light green
*Z* = 4	0.17 × 0.14 × 0.07 mm

### Data collection

**Table:**

Bruker APEXII CCD diffractometer	2483 reflections with *I* > 2σ(*I*)
φ and ω scans	*R*_int_ = 0.108
Absorption correction: multi-scan (SADABS; Bruker, 2012)	θ_max_ = 26.5°, θ_min_ = 2.0°
*T*_min_ = 0.683, *T*_max_ = 0.745	*h* = −25→22
22477 measured reflections	*k* = −12→15
4670 independent reflections	*l* = −24→24

### Refinement

**Table:**

Refinement on *F*^2^	2 restraints
Least-squares matrix: full	Hydrogen site location: mixed
*R*[*F*^2^ > 2σ(*F*^2^)] = 0.061	H atoms treated by a mixture of independent and constrained refinement
*wR*(*F*^2^) = 0.125	*w* = 1/[σ^2^(*F*_o_^2^) + (0.0372*P*)^2^ + 4.4221*P*] where *P* = (*F*_o_^2^ + 2*F*_c_^2^)/3
*S* = 1.00	(Δ/σ)_max_ < 0.001
4670 reflections	Δρ_max_ = 0.36 e Å^−^^3^
310 parameters	Δρ_min_ = −0.39 e Å^−^^3^

### Special details

**Table:**

Geometry. All esds (except the esd in the dihedral angle between two l.s. planes) are estimated using the full covariance matrix. The cell esds are taken into account individually in the estimation of esds in distances, angles and torsion angles; correlations between esds in cell parameters are only used when they are defined by crystal symmetry. An approximate (isotropic) treatment of cell esds is used for estimating esds involving l.s. planes.

### Fractional atomic coordinates and isotropic or equivalent isotropic displacement parameters (Å^2^)

**Table:**

	*x*	*y*	*z*	*U*_iso_*/*U*_eq_	
Ni	0.5000	0.13563 (5)	0.2500	0.0258 (2)	
N1	0.44888 (16)	0.2530 (2)	0.28779 (16)	0.0277 (7)	
N2	0.56997 (15)	0.1514 (2)	0.35984 (15)	0.0258 (7)	
N3	0.44732 (16)	0.0053 (2)	0.27341 (15)	0.0251 (7)	
C1	0.3878 (2)	0.3033 (3)	0.2479 (2)	0.0358 (10)	
H1	0.3624	0.2810	0.1981	0.043*	
C2	0.3600 (2)	0.3857 (3)	0.2755 (2)	0.0412 (11)	
H2	0.3160	0.4185	0.2459	0.049*	
C3	0.3976 (2)	0.4192 (3)	0.3472 (2)	0.0424 (11)	
H3	0.3802	0.4766	0.3675	0.051*	
C4	0.4604 (2)	0.3688 (3)	0.3890 (2)	0.0375 (10)	
H4	0.4868	0.3911	0.4386	0.045*	
C5	0.48510 (19)	0.2855 (3)	0.3585 (2)	0.0272 (9)	
C6	0.55264 (19)	0.2279 (3)	0.3992 (2)	0.0250 (9)	
C7	0.5973 (2)	0.2508 (3)	0.4718 (2)	0.0336 (10)	
H7	0.5844	0.3045	0.4989	0.040*	
C8	0.6604 (2)	0.1949 (3)	0.5044 (2)	0.0353 (10)	
H8	0.6916	0.2097	0.5540	0.042*	
C9	0.6777 (2)	0.1174 (3)	0.4641 (2)	0.0344 (10)	
H9	0.7210	0.0784	0.4852	0.041*	
C10	0.6310 (2)	0.0976 (3)	0.3927 (2)	0.0319 (10)	
H10	0.6427	0.0430	0.3653	0.038*	
C11	0.3952 (2)	0.0107 (3)	0.29930 (19)	0.0335 (10)	
H11	0.3776	0.0795	0.3050	0.040*	
C12	0.3661 (2)	−0.0790 (3)	0.3179 (2)	0.0380 (10)	
H12	0.3289	−0.0720	0.3355	0.046*	
C13	0.3915 (2)	−0.1782 (3)	0.3106 (2)	0.0391 (11)	
H13	0.3732	−0.2411	0.3243	0.047*	
C14	0.4440 (2)	−0.1851 (3)	0.2830 (2)	0.0354 (10)	
H14	0.4618	−0.2533	0.2766	0.042*	
C15	0.47114 (19)	−0.0920 (3)	0.26463 (18)	0.0273 (9)	
N4	0.2358 (2)	0.9371 (3)	0.0957 (2)	0.0518 (10)	
N5	0.25094 (19)	0.6205 (3)	0.1895 (2)	0.0578 (11)	
N6	0.3282 (2)	0.4535 (3)	0.10158 (19)	0.0561 (11)	
N7	0.5030 (2)	0.6277 (3)	0.07484 (19)	0.0488 (9)	
O1	0.38199 (14)	0.8243 (2)	0.08018 (14)	0.0389 (7)	
C16	0.3536 (2)	0.7331 (3)	0.0959 (2)	0.0341 (10)	
C17	0.2966 (2)	0.7526 (3)	0.1182 (2)	0.0345 (10)	
C18	0.2635 (2)	0.8550 (4)	0.1055 (2)	0.0385 (11)	
C19	0.2720 (2)	0.6775 (4)	0.1570 (2)	0.0435 (11)	
C20	0.3857 (2)	0.6355 (3)	0.0944 (2)	0.0378 (10)	
C21	0.3536 (2)	0.5348 (4)	0.0987 (2)	0.0412 (11)	
C22	0.4508 (2)	0.6307 (3)	0.0835 (2)	0.0379 (10)	
C23	0.3802 (2)	0.8371 (3)	0.0057 (2)	0.0412 (11)	
H23A	0.3362	0.8755	−0.0257	0.049*	
H23B	0.3803	0.7657	−0.0166	0.049*	
C24	0.4438 (2)	0.8993 (3)	0.0101 (2)	0.0489 (12)	
H24A	0.4451	0.9676	0.0355	0.073*	
H24B	0.4414	0.9136	−0.0400	0.073*	
H24C	0.4870	0.8579	0.0377	0.073*	
O2	0.2745 (2)	0.6530 (3)	0.3463 (2)	0.0829 (11)	
H25	0.274 (3)	0.634 (4)	0.3041 (15)	0.099*	
H26	0.271 (3)	0.592 (2)	0.365 (3)	0.099*	

### Atomic displacement parameters (Å^2^)

**Table:**

	*U*^11^	*U*^22^	*U*^33^	*U*^12^	*U*^13^	*U*^23^
Ni	0.0223 (4)	0.0255 (4)	0.0259 (4)	0.000	0.0053 (3)	0.000
N1	0.0243 (18)	0.0273 (18)	0.0285 (19)	0.0033 (16)	0.0070 (16)	0.0031 (15)
N2	0.0226 (17)	0.0255 (18)	0.0262 (17)	0.0024 (16)	0.0059 (15)	0.0002 (15)
N3	0.0239 (18)	0.0253 (18)	0.0218 (17)	−0.0001 (16)	0.0040 (15)	0.0005 (14)
C1	0.030 (2)	0.036 (2)	0.037 (2)	0.007 (2)	0.008 (2)	0.005 (2)
C2	0.034 (2)	0.039 (3)	0.052 (3)	0.013 (2)	0.018 (2)	0.007 (2)
C3	0.041 (3)	0.036 (3)	0.053 (3)	0.011 (2)	0.022 (2)	−0.005 (2)
C4	0.039 (3)	0.038 (2)	0.037 (2)	0.005 (2)	0.015 (2)	−0.004 (2)
C5	0.024 (2)	0.026 (2)	0.033 (2)	0.0029 (19)	0.0130 (19)	0.0025 (19)
C6	0.028 (2)	0.020 (2)	0.027 (2)	−0.0019 (18)	0.0105 (19)	0.0012 (17)
C7	0.036 (3)	0.035 (2)	0.030 (2)	0.003 (2)	0.013 (2)	−0.003 (2)
C8	0.032 (2)	0.041 (3)	0.024 (2)	−0.004 (2)	0.002 (2)	−0.001 (2)
C9	0.022 (2)	0.039 (3)	0.032 (2)	0.004 (2)	−0.0004 (19)	0.003 (2)
C10	0.025 (2)	0.032 (2)	0.033 (2)	0.003 (2)	0.005 (2)	−0.0005 (19)
C11	0.029 (2)	0.039 (3)	0.031 (2)	0.003 (2)	0.008 (2)	0.005 (2)
C12	0.027 (2)	0.045 (3)	0.038 (3)	−0.001 (2)	0.009 (2)	0.009 (2)
C13	0.034 (3)	0.036 (3)	0.038 (3)	−0.011 (2)	0.003 (2)	0.008 (2)
C14	0.036 (3)	0.031 (2)	0.032 (2)	−0.003 (2)	0.005 (2)	−0.001 (2)
C15	0.023 (2)	0.029 (2)	0.020 (2)	−0.0042 (19)	−0.0026 (17)	−0.0011 (17)
N4	0.048 (3)	0.047 (3)	0.067 (3)	−0.006 (2)	0.030 (2)	−0.003 (2)
N5	0.055 (3)	0.067 (3)	0.056 (3)	−0.010 (2)	0.027 (2)	0.013 (2)
N6	0.065 (3)	0.044 (2)	0.046 (2)	−0.021 (2)	0.007 (2)	−0.002 (2)
N7	0.051 (2)	0.043 (2)	0.053 (2)	0.003 (2)	0.020 (2)	−0.0022 (19)
O1	0.0416 (17)	0.0363 (17)	0.0430 (17)	−0.0162 (14)	0.0211 (14)	−0.0076 (13)
C16	0.035 (3)	0.037 (3)	0.022 (2)	−0.015 (2)	0.003 (2)	−0.0040 (19)
C17	0.031 (2)	0.035 (3)	0.031 (2)	−0.015 (2)	0.005 (2)	0.000 (2)
C18	0.033 (3)	0.049 (3)	0.035 (2)	−0.016 (3)	0.014 (2)	−0.002 (2)
C19	0.034 (3)	0.054 (3)	0.038 (3)	−0.013 (2)	0.008 (2)	−0.004 (2)
C20	0.043 (3)	0.035 (2)	0.034 (2)	−0.009 (3)	0.013 (2)	−0.004 (2)
C21	0.041 (3)	0.047 (3)	0.027 (2)	−0.008 (3)	0.002 (2)	−0.003 (2)
C22	0.050 (3)	0.031 (2)	0.029 (2)	−0.004 (3)	0.011 (2)	−0.003 (2)
C23	0.043 (3)	0.045 (3)	0.030 (2)	−0.006 (2)	0.008 (2)	0.003 (2)
C24	0.050 (3)	0.052 (3)	0.048 (3)	−0.010 (2)	0.023 (2)	0.002 (2)
O2	0.112 (3)	0.070 (3)	0.078 (3)	−0.017 (3)	0.049 (3)	−0.009 (2)

### Geometric parameters (Å, º)

**Table:**

Ni—N2	2.077 (3)	C11—C12	1.376 (5)
Ni—N2^i^	2.077 (3)	C11—H11	0.9500
Ni—N1	2.088 (3)	C12—C13	1.366 (5)
Ni—N1^i^	2.088 (3)	C12—H12	0.9500
Ni—N3	2.090 (3)	C13—C14	1.373 (5)
Ni—N3^i^	2.090 (3)	C13—H13	0.9500
N1—C1	1.339 (4)	C14—C15	1.389 (5)
N1—C5	1.353 (4)	C14—H14	0.9500
N2—C10	1.335 (4)	C15—C15^i^	1.493 (7)
N2—C6	1.354 (4)	N4—C18	1.146 (5)
N3—C15	1.339 (4)	N5—C19	1.141 (5)
N3—C11	1.343 (4)	N6—C21	1.146 (5)
C1—C2	1.379 (5)	N7—C22	1.139 (5)
C1—H1	0.9500	O1—C16	1.361 (4)
C2—C3	1.376 (5)	O1—C23	1.453 (4)
C2—H2	0.9500	C16—C20	1.384 (5)
C3—C4	1.373 (5)	C16—C17	1.409 (5)
C3—H3	0.9500	C17—C19	1.413 (5)
C4—C5	1.384 (5)	C17—C18	1.417 (6)
C4—H4	0.9500	C20—C22	1.422 (6)
C5—C6	1.479 (5)	C20—C21	1.431 (6)
C6—C7	1.388 (5)	C23—C24	1.482 (5)
C7—C8	1.381 (5)	C23—H23A	0.9900
C7—H7	0.9500	C23—H23B	0.9900
C8—C9	1.374 (5)	C24—H24A	0.9800
C8—H8	0.9500	C24—H24B	0.9800
C9—C10	1.374 (5)	C24—H24C	0.9800
C9—H9	0.9500	O2—H25	0.856 (10)
C10—H10	0.9500	O2—H26	0.860 (10)
			
N1—Ni—N2	78.64 (12)	C8—C9—C10	118.7 (4)
N1^i^—Ni—N2	93.73 (11)	C8—C9—H9	120.7
N1^i^—Ni—N2	93.73 (11)	C10—C9—H9	120.7
N2^i^—Ni—N1^i^	78.64 (12)	N2—C10—C9	123.2 (3)
N1—Ni—N1^i^	91.20 (16)	N2—C10—H10	118.4
N1^i^—Ni—N3	171.36 (11)	C9—C10—H10	118.4
N1—Ni—N3^i^	171.36 (11)	N3—C11—C12	122.8 (4)
N2—Ni—N2^i^	169.18 (16)	N3—C11—H11	118.6
N2—Ni—N3	92.87 (11)	C12—C11—H11	118.6
N2^i^—Ni—N3	95.51 (11)	C13—C12—C11	119.1 (4)
N1—Ni—N3	95.55 (11)	C13—C12—H12	120.5
N2—Ni—N3^i^	95.52 (11)	C11—C12—H12	120.5
N2^i^—Ni—N3^i^	92.87 (11)	C12—C13—C14	118.8 (4)
N1^i^—Ni—N3^i^	95.55 (11)	C12—C13—H13	120.6
N3—Ni—N3^i^	78.26 (16)	C14—C13—H13	120.6
C1—N1—C5	118.2 (3)	C13—C14—C15	119.8 (4)
C1—N1—Ni	126.6 (3)	C13—C14—H14	120.1
C5—N1—Ni	115.0 (2)	C15—C14—H14	120.1
C10—N2—C6	118.5 (3)	N3—C15—C14	121.3 (3)
C10—N2—Ni	126.0 (2)	N3—C15—C15^i^	115.3 (2)
C6—N2—Ni	115.5 (2)	C14—C15—C15^i^	123.4 (2)
C15—N3—C11	118.2 (3)	C16—O1—C23	118.1 (3)
C15—N3—Ni	115.5 (2)	O1—C16—C20	118.7 (3)
C11—N3—Ni	126.2 (3)	O1—C16—C17	113.5 (4)
N1—C1—C2	123.2 (4)	C20—C16—C17	127.6 (4)
N1—C1—H1	118.4	C16—C17—C19	124.0 (4)
C2—C1—H1	118.4	C16—C17—C18	119.5 (3)
C3—C2—C1	118.3 (4)	C19—C17—C18	116.4 (4)
C3—C2—H2	120.8	N4—C18—C17	178.8 (4)
C1—C2—H2	120.8	N5—C19—C17	177.1 (5)
C4—C3—C2	119.4 (4)	C16—C20—C22	121.0 (4)
C4—C3—H3	120.3	C16—C20—C21	122.4 (4)
C2—C3—H3	120.3	C22—C20—C21	116.5 (4)
C3—C4—C5	119.5 (4)	N6—C21—C20	179.0 (5)
C3—C4—H4	120.2	N7—C22—C20	179.4 (5)
C5—C4—H4	120.2	O1—C23—C24	108.5 (3)
N1—C5—C4	121.4 (3)	O1—C23—H23A	110.0
N1—C5—C6	115.5 (3)	C24—C23—H23A	110.0
C4—C5—C6	123.1 (3)	O1—C23—H23B	110.0
N2—C6—C7	121.0 (3)	C24—C23—H23B	110.0
N2—C6—C5	115.2 (3)	H23A—C23—H23B	108.4
C7—C6—C5	123.7 (3)	C23—C24—H24A	109.5
C8—C7—C6	119.5 (3)	C23—C24—H24B	109.5
C8—C7—H7	120.3	H24A—C24—H24B	109.5
C6—C7—H7	120.3	C23—C24—H24C	109.5
C9—C8—C7	119.1 (4)	H24A—C24—H24C	109.5
C9—C8—H8	120.4	H24B—C24—H24C	109.5
C7—C8—H8	120.4	H25—O2—H26	102 (5)
			
C5—N1—C1—C2	−0.4 (5)	C3—C4—C5—C6	179.2 (3)
Ni—N1—C1—C2	−175.1 (3)	C10—N2—C6—C7	0.4 (5)
N1—C1—C2—C3	1.2 (6)	Ni—N2—C6—C7	−176.1 (3)
C1—C2—C3—C4	−1.1 (6)	C10—N2—C6—C5	178.7 (3)
C2—C3—C4—C5	0.2 (6)	Ni—N2—C6—C5	2.2 (4)
C1—N1—C5—C4	−0.5 (5)	N1—C5—C6—N2	1.1 (4)
Ni—N1—C5—C4	174.8 (3)	C4—C5—C6—N2	−177.5 (3)
C1—N1—C5—C6	−179.2 (3)	N1—C5—C6—C7	179.4 (3)
Ni—N1—C5—C6	−3.8 (4)	C4—C5—C6—C7	0.8 (5)
C3—C4—C5—N1	0.6 (5)	N2—C6—C7—C8	0.3 (5)

Symmetry code: (i) −*x*+1, *y*, −*z*+1/2.

### Hydrogen-bond geometry (Å, º)

*Cg*1 is the centroid of the N2/C6–C10 ring.

**Table:**

*D*—H···*A*	*D*—H	H···*A*	*D*···*A*	*D*—H···*A*
O2—H25···N5	0.86	2.11	2.945 (5)	164
O2—H26···N4^ii^	0.86	2.10	2.955 (5)	175
C24—H24*A*···*Cg*1^iii^	0.98	3.01	3.921	156

Symmetry codes: (ii) −*x*+1/2, *y*−1/2, −*z*+1/2; (iii) −*x*+1/2, −*y*+3/2, −*z*.

### Selected geometry parameters (Å,°)

**Table:**

Ni-N1	2.088 (3)
Ni-N2	2.077 (3)
Ni-N3	2.090 (3)
N1-Ni-N2	78.64 (12)
N1-Ni-N3	95.55 (11)
N2-Ni-N3	92.87 (11)

## References

[bb1] Addala, A., Setifi, F., Kottrup, K. G., Glidewell, C., Setifi, Z., Smith, G. & Reedijk, J. (2015). *Polyhedron*, **87**, 307–310.

[bb2] Atmani, C., Setifi, F., Benmansour, S., Triki, S., Marchivie, M., Salaün, J.-Y. & Gómez-García, C. J. (2008). *Inorg. Chem. Commun.* **11**, 921–924.

[bb3] Benmansour, S., Atmani, C., Setifi, F., Triki, S., Marchivie, M. & Gómez-García, C. J. (2010). *Coord. Chem. Rev.* **254**, 1468–1478.

[bb4] Benmansour, S., Setifi, F., Gómez-García, C. J., Triki, S., Coronado, E. & Salaün, J. (2008). *J. Mol. Struct.* **890**, 255–262.

[bb5] Benmansour, S., Setifi, F., Triki, S., Salaün, J.-Y., Vandevelde, F., Sala-Pala, J., Gómez-García, C. J. & Roisnel, T. (2007). *Eur. J. Inorg. Chem.* pp. 186–194.

[bb6] Bruker (2012). *APEX2*, *SAINT* and *SADABS*. Bruker AXS Inc., Madison, Wisconsin, USA.

[bb7] Freire, E., Baggio, S., Mombrú, A. & Baggio, R. (2000). *Acta Cryst.* C**56**, 541–543. 10.1107/S0108270100001414 10851608

[bb8] Gaamoune, B., Setifi, Z., Beghidja, A., El-Ghozzi, M., Setifi, F. & Avignant, D. (2010). *Acta Cryst.* E**66**, m1044–m1045. 10.1107/S1600536810029752 PMC3007362 21588113

[bb9] Groom, C. R., Bruno, I. J., Lightfoot, M. P. & Ward, S. C. (2016). *Acta Cryst.* B**72**, 171–179. 10.1107/S2052520616003954 PMC4822653 27048719

[bb10] Guillot, B., Enrique, E., Huder, L. & Jelsch, C. (2014). *Acta Cryst.* A**70**, C279.

[bb11] Jelsch, C., Ejsmont, K. & Huder, L. (2014). *IUCrJ*, **1**, 119–128. 10.1107/S2052252514003327 PMC4062089 25075328

[bb12] McKinnon, J. J., Jayatilaka, D. & Spackman, M. A. (2007). *Chem. Commun.* pp. 3814–3816. 10.1039/b704980c 18217656

[bb13] Miyazaki, A., Okabe, K., Enoki, T., Setifi, F., Golhen, S., Ouahab, L., Toita, T. & Yamada, J. (2003). *Synth. Met.* **137**, 1195–1196.

[bb14] Setifi, F., Konieczny, P., Glidewell, C., Arefian, M., Pelka, R., Setifi, Z. & Mirzaei, M. (2017). *J. Mol. Struct.* **1149**, 149–154.

[bb15] Setifi, F., Valkonen, A., Setifi, Z., Nummelin, S., Touzani, R. & Glidewell, C. (2016). *Acta Cryst.* E**72**, 1246–1250. 10.1107/S2056989016012160 PMC5120698 27920908

[bb16] Setifi, Z., Domasevitch, K. V., Setifi, F., Mach, P., Ng, S. W., Petříček, V. & Dušek, M. (2013*a*). *Acta Cryst.* C**69**, 1351–1356. 10.1107/S0108270113027108 24192188

[bb17] Setifi, Z., Gaamoune, B., Stoeckli-Evans, H., Rouag, D.-A. & Setifi, F. (2010). *Acta Cryst.* C**66**, m286–m289. 10.1107/S010827011003578X 20921605

[bb18] Setifi, Z., Lehchili, F., Setifi, F., Beghidja, A., Ng, S. W. & Glidewell, C. (2014*b*). *Acta Cryst.* C**70**, 338–341. 10.1107/S2053229614004379 24594730

[bb19] Setifi, Z., Setifi, F., Boughzala, H., Beghidja, A. & Glidewell, C. (2014*c*). *Acta Cryst.* C**70**, 465–469. 10.1107/S2053229614008092 24816015

[bb20] Setifi, Z., Setifi, F., El Ammari, L., El-Ghozzi, M., Sopková-de Oliveira Santos, J., Merazig, H. & Glidewell, C. (2014*a*). *Acta Cryst.* C**70**, 19–22. 10.1107/S2053229613032804 24399219

[bb21] Setifi, Z., Setifi, F., Ng, S. W., Oudahmane, A., El-Ghozzi, M. & Avignant, D. (2013*b*). *Acta Cryst.* E**69**, m12–m13. 10.1107/S1600536812048611 PMC3588335 23476315

[bb22] Setifi, Z., Valkonen, A., Fernandes, M. A., Nummelin, S., Boughzala, H., Setifi, F. & Glidewell, C. (2015). *Acta Cryst.* E**71**, 509–515. 10.1107/S2056989015007306 PMC4420140 25995868

[bb23] Shannon, R. D. & Prewitt, C. T. (1969). *Acta Cryst.* B**25**, 925–946.

[bb24] Sheldrick, G. M. (2008). *Acta Cryst.* A**64**, 112–122. 10.1107/S0108767307043930 18156677

[bb25] Sheldrick, G. M. (2015). *Acta Cryst.* C**71**, 3–8.

[bb26] Su, Z.-H., Zhou, B.-B., Zhao, Z.-F. & Zhang, Y.-N. (2007). *Acta Cryst.* E**63**, m1206–m1207.

[bb27] Thétiot, F., Triki, S. & Sala Pala, J. (2003). *Polyhedron*, **22**, 1837–1843.

[bb28] Westrip, S. P. (2010). *J. Appl. Cryst.* **43**, 920–925.

[bb29] Wolff, S. K., Grimwood, D. J., McKinnon, J. J., Turner, M. J., Jayatilaka, D. & Spackman, M. A. (2012). Crystal Explorer. University of Western Australia.

[bb30] Yang, G.-Y., Gao, D.-W., Chen, Y., Xu, J.-Q., Zeng, Q.-X., Sun, H.-R., Pei, Z.-W., Su, Q., Xing, Y., Ling, Y.-H. & Jia, H.-Q. (1998). *Acta Cryst.* C**54**, 616–618.

[bb31] Yuste, C., Bentama, A., Marino, N., Armentano, D., Setifi, F., Triki, S., Lloret, F. & Julve, M. (2009). *Polyhedron*, **28**, 1287–1294.

